# Psychotherapies and psychological support for individuals facing psychological distress during the COVID-19 pandemic: A scoping review

**DOI:** 10.1371/journal.pone.0318192

**Published:** 2025-04-01

**Authors:** Mao Yagihashi, Atsushi Sakuma, Michio Murakami

**Affiliations:** 1 Division of Scientific Information and Public Policy, Center for Infectious Diseases Education and Research (CiDER), Osaka University, Suita, Japan; 2 Department of Psychiatry, Sendai Medical Center, Sendai, Japan; Jordan University of Science and Technology, Jordan

## Abstract

In this scoping review, we investigated the psychotherapies and psychological support provided during the coronavirus disease 2019 (COVID-19) pandemic to clarify its recipients and the methods employed, among other characteristics. We used Scopus and PubMed as the two search engines and employed the following search terms: “COVID*” AND (“psychotherap*” OR “psychological support*”) AND “psychological distress*.” The first search was conducted on October 18, 2022, by reviewing search engines and conducting a manual search. It yielded 154 articles, of which 18 finally met the eligibility criteria after the second round of screening. The distribution of the participants in the intervention studies included in this review was diverse, including the general population, patients with COVID-19, and patients who had recovered from COVID-19. The implementation of psychotherapy was not limited to more advanced countries in psychiatry, indicating the broader reach of these interventions. Psychotherapy includes various methods, including cognitive behavioral therapy, acceptance commitment therapy, mindfulness, supportive care, virtual reality therapy, and online educational content via apps. The rise of new technologies may have increased the replacement rate of human therapists. In addition, the use of remote interventions was more common than that of face-to-face interventions. It is necessary to monitor whether the technologies and methods used for the first time during the pandemic will continue to be used in the future. Therefore, further research is needed to compare the effectiveness of remote randomized controlled trials with conventional face-to-face randomized controlled trials. Finally, most of those providing psychotherapies and psychological support in the studies included in this review were not doctors or psychologists.

## Introduction

The deterioration of mental health caused by the coronavirus disease 2019 (COVID-19) is a public health concern globally [1]. The exacerbation of psychiatric symptoms amid the COVID-19 spread can be attributed to the bio-psycho-social model [2], as is typically observed in the development of psychiatric symptoms. This model includes biological factors such as physical pain and its sequelae, that is, lingering symptoms caused by COVID-19 infection [3]; psychological factors such as isolation due to behavioral restrictions and fear of infection [4], a sense of self-inflicted guilt for having been infected [5], and discrimination against infected people or people with specific backgrounds [6]; and social factors such as lockdown and refraining from activities [7] and economic deprivation caused by lockdowns and business restrictions [8]. Disaster psychiatry studies that focus on pandemics have shown that people may choose to avoid seeking psychological help during the acute phase of an infectious threat and that many mental health disorders will subsequently surface as the infectious threat decreases because requests for mental healthcare and support services will then increase rapidly [9].

While some studies have investigated this topic, more attention should be paid to psychological support for those who have recovered from the acute symptoms of COVID-19 but still suffer from chronic symptoms. For example, an early review of healthcare workers’ mental health during the COVID-19 pandemic reported six intervention studies on this topic; however, none of these six studies reported the effectiveness of the examined interventions [10]. Another study reviewed psychological support interventions for healthcare professionals and informal caregivers (i.e., family members and others close to them) during the COVID-19 pandemic [11]. Additionally, a narrative review of interventions for family caregivers of patients with amyotrophic lateral sclerosis reported their experience using telemedicine [12]. However, as of October 8, 2022 (searched again to confirm before submitting on July 5, 2023), none of the 21 reviews found by searching Scopus using the terms “COVID,” “distress,” and “psychological support” had summarized the types of psychotherapies and psychological support offered to diverse audiences or the means through which they were delivered.

Determining what types of psychotherapies and psychological support should be provided for those affected by COVID-19 as well as by emerging infectious diseases in the future is crucial. Reviewing the literature on the psychotherapies and psychological support provided during the pandemic would allow the public to reflect on the support available at that time and help healthcare professionals and medical staff review the support they could provide. A few of the psychotherapies or psychological support carried out in medical settings have thus far been examined in academic studies; the papers published on this topic are significant and must be used effectively to shed necessary light on global trends and perspectives. This would be useful not only for COVID-19 but also for intervention research after future infectious disease outbreaks and disasters. Based on the foregoing, we conducted a scoping review to clarify the types of psychotherapies and psychological support provided during the COVID-19 pandemic and the providers.

## Materials and methods

**Fig 1:**
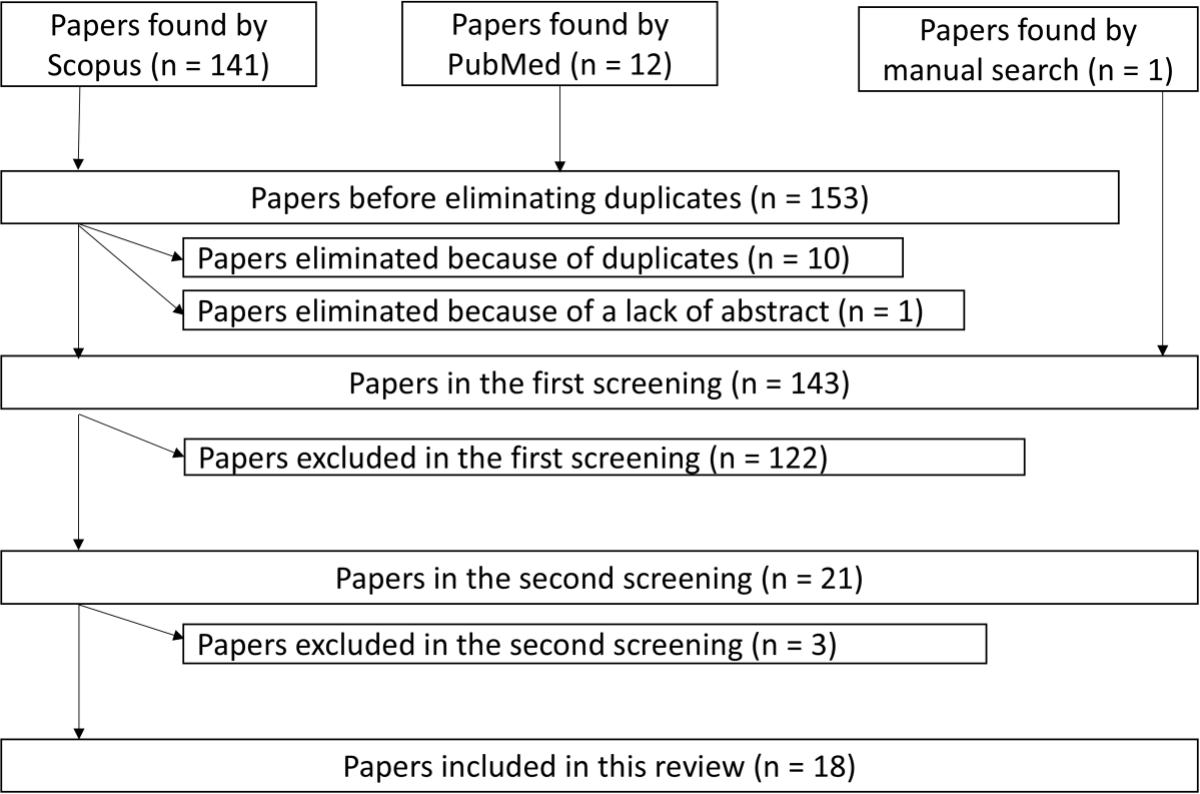
Screening process in this study. Note. We conducted the first screening for the paper found by a manual search in accordance with the protocol described in this study, but it did not meet our selection criteria and was removed.

Of the manifold review methods (i.e., literature review, scoping review, systematic review, umbrella review), scoping reviews are a relatively new method of identifying knowledge gaps and are often conducted as a preliminary step to systematic reviews. While similar to systematic reviews, scoping reviews do not evaluate targeted studies or integrate their results. The methodology for scoping reviews was first proposed by Arksey and O’Malley in 2005 [13] and later developed by Levac et al. [14] in 2010. In 2015, the JBI scoping review methodology group developed guidelines for conducting scoping reviews [15]. In 2018, to promote transparency when using this methodology, Tricco et al. [16] proposed the Preferred Reporting Items for Systematic Reviews and Meta-Analysis Extension for Scoping Reviews (PRISMA-ScR) reporting guidelines for scoping reviews. In this study, we chose a scoping review as our methodology because it was the most suitable to clarify the types of psychotherapies and psychological support provided during the COVID-19 pandemic. Therefore, this study was conducted according to the PRISMA-ScR guidelines [16]. The review was conducted in four phases: identification, screening, eligibility assessment, and final synthesis (Fig 1).

### Identification

We conducted our search on October 18, 2022. Since the objective was to determine the types of psychotherapies and psychological support provided for psychological distress during the COVID-19 pandemic, the primary keywords were “COVID-19,” “psychotherapy” (or) “psychological support,” and “psychological distress.” To be able to search and access all targeted papers without omission, we searched two search engines (PubMed and Scopus) following previous research [17]. First, PubMed is a paper search tool developed by the National Library of Medicine in the United States, part of the National Institutes of Health. Owing to its high profile and practical advantages, it is the most frequently used source of information in the biomedical field. In particular, PubMed allows researchers to search and view articles from an early stage (i.e., from the first edition). Second, Scopus is a journal search tool run by Elsevier in Europe. It can search a wider range of journal fields than PubMed; however, articles cannot be viewed until they are officially published. In addition, the papers listed are limited to those published after 1995. However, as we studied psychotherapies and psychological support conducted during the COVID-19 pandemic (i.e., from 2019), this was not a concern. Therefore, to conduct a thorough and accurate search for recent papers, we used both databases.

In this study, the inclusion criteria were as follows: the article had to be an original paper or review article, its full text had to be available (not just the abstract), and it had to be written in English. Protocol articles were also included among the articles obtained by the search engine but were excluded from the screening. The search formula for Scopus was the following: TITLE-ABS-KEY (“COVID*” AND (“psychotherap*” OR “psychological support*”) AND “psychological distress*”) AND (LIMIT-TO (DOCTYPE,”ar”) OR LIMIT-TO (DOCTYPE,”re”)) AND (LIMIT-TO (LANGUAGE,”English”))). The search formula for PubMed was as follows: ((“covid*”[Title/Abstract] OR “COVID-19”[MeSH Terms]) AND (“psychotherap*”[Title/Abstract] OR “psychological support*”[Title/Abstract]) AND (“ Psychological Distress *”[Title/Abstract] OR “Psychological Distress”[MeSH Terms])) AND ((ffrft[Filter]) AND (fha[Filter]) AND (clinicaltrial[Filter] OR meta-analysis[Filter] OR randomizedcontrolledtrial[Filter] OR review[Filter] OR systematicreview[Filter]) AND (fft[Filter])).

### Screening

The screening procedure was conducted in two stages (Fig 1). During the first stage on October 18, 2022, the search yielded 153 entries (141 from Scopus, 12 from PubMed). In addition, one entry was added through manual search [18]. All these entries (n =  154) were output using Endnote reference management (Endnote X8, Clarivate Analytics). Duplicates (n =  10) and those without abstracts (n =  1) were removed according to the inclusion criteria. In the first screening, two independent experts, M.Y. (major in clinical psychology) and M.M. (major in risk science), determined whether the inclusion criteria were met based on the titles and abstracts. The results were integrated through discussion. The kappa coefficient of agreement was 0.68. When the judgments were not in accordance with the two independent experts mentioned above, a third expert, A.S. (major in psychiatry, especially liaison psychiatry), was consulted. A total of 122 papers were excluded in the first screening stage, including the paper added by manual search (n =  1). Hence, 21 papers remained eligible for the second screening stage.

In the second stage, all 21 papers were obtained by manual search. Two independent experts (M.M. and A.S.) read half of the articles and M.Y. read them all. Three studies were subsequently excluded, leaving 18 studies in the final review. We reconfirmed that these met the inclusion criteria for this study and itemized the content according to the intervention participants, number of participants, countries in which the psychotherapies and psychological support were provided, target population for psychotherapies and psychological support, types and means of this support, support provider, and means of the measures used.

- Intervention participants.

Participants were classified according to the descriptions provided in the articles, allowing for duplicate responses.

- Number of participants.

The total number of participants and number of intervention and control groups were recorded according to each study design.

- Countries.

The countries in which the interventions were implemented were documented and categorized based on the descriptions in the articles.

- Types of psychotherapies and psychological support.

Classifications were made broadly considering the real-world settings of the interventions, and duplicate responses were allowed. Humanistic psychology is a form of psychology developed in the United States in the 1960s [19]. In particular, client-centered therapy advocated by Rogers is well known, and the three core conditions (congruence (authenticity), unconditional positive regard, and empathic understanding) are the basic attitudes required of those involved in psychological support, such as therapists and counselors. Cognitive behavioral therapy (CBT) primarily comprises cognitive therapy and behavioral therapy. Based on these, psychotherapies, known as third-generation therapies, such as acceptance commitment therapy (ACT) and mindfulness, have emerged since the 1990s [19]. Various versions of these therapies specialize in symptoms or diseases. In this study, we broadly classified them according to the descriptions in the papers (e.g., simplified CBT - Insomnia (S - CBTI) was classified as CBT). Telephone support was also classified according to the theory or therapy on which it was based if a description was provided. If no description was provided, we classified it as telephone support. Furthermore, for intervention studies divided into control and intervention groups, support for the control group was not included (e.g., distribution of newsletters and provision of regular support).

- Types of interventions.

The following items were classified: validation of feasibility, randomized controlled trials (RCTs), established existing interventions, and others. Duplicate responses were allowed.

- Means of psychotherapies and psychological support.

Means of support were classified as remote, face-to-face, and others. Remote communication included both voice-only, including conventional telephone calls and calls made online, and video calls but was defined as remote when real-time two-way communication was possible. Duplicate responses were allowed.

- Providers.

Providers included medical doctors, psychologists/counselors, and others. To maintain consistency, “psychologist” and “counselor” were considered to be identical for classification purposes, acknowledging that the definitions may vary by country. Duplicate responses were allowed.

- Means of the measures used

These were written according to the descriptions in the studies. Duplicate responses were allowed.

### Eligibility assessment and inclusion

After the second screening, if the two independent researchers disagreed on the results, the three experts discussed and reached a consensus. Consequently, 18 papers were included in this study [14-31]. The paper’s content was compiled by M.Y. and subsequently reviewed and edited by M.M. and A.S.

## Results

**Fig 2:**
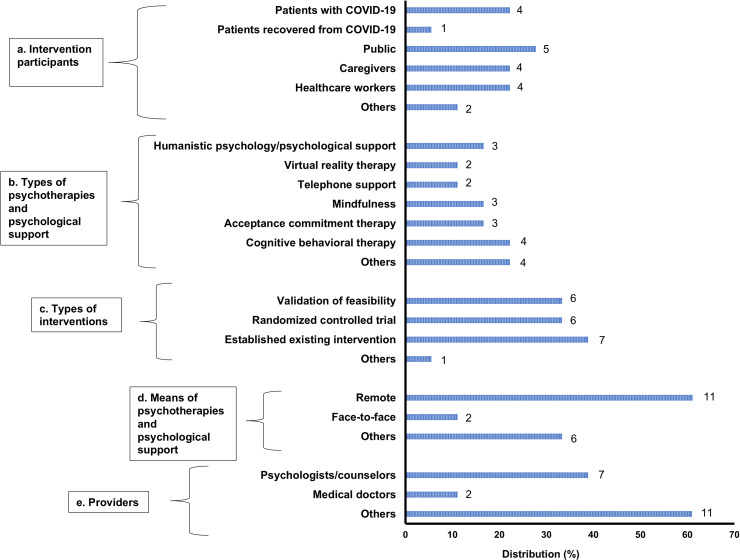
Distribution of the survey items in the subject papers.

Table 1 summarizes the papers included in this review, and Fig 2 shows the distribution of each item.

**Table 1:** Overview of the papers included in this study.

Ref.	Period	Country	Intervention participants	Number of participants; interventions (I) vs. controls (C)	Types of interventions	Types of support	Means of support	Provider	Means of the measures used	Paper summary
[20]	Assessments were between September 2019 and June 2020	Jordan	Refugees	410 (I: 204, C: 206)	RCT	Usual care vs. Problem Management Plus	Face-to-face	Trained but non-specialist	PCL HSCL	This study examined the psychological impact of the pandemic on the mental health of Syrian refugees living in the Azraq camp in Jordan. Severe post-traumatic stress disorder symptoms were less common among the refugees assessed during the pandemic than those assessed before the pandemic.
[21]	Participants were recruited between April 14 and May 30, 2020	Oman	Public	46 (I: 22, C: 24)	RCT	Autonomic weekly newsletter containing self-help therapy vs. therapy based on the principles of CBT and ACT	Remote	Psychologist/counselor	PHQ GAD	This study compared the efficacy of therapist-guided online therapy (CBT + ACT) with self-help therapy by weekly newsletters for people living in Oman during the COVID-19 pandemic. The levels of anxiety and depression were reduced in both groups; however, the reduction was higher in the intervention group than in the control group.
[22]	Participants were recruited between February 21 and July 31, 2020	Italy	Patients after recovery from COVID-19	264 (I: 16)	Established existing intervention	Supportive psychotherapy based on humanistic psychology	Remote	Psychologist/counselor	HADS IES-R	This study described the psychological status of patients recovering from COVID-19. In particular, it focused on anxiety, depressive symptoms, and post-traumatic stress. The results showed that patients reported anxiety (28%), depression (17%), and post-traumatic stress (36.4%). In total, 13.8% of patients received psychological visits and 6.1% received psychological support. Patients who had recovered from COVID-19 reported negative mental health outcomes in the months following discharge.
[23]	Not described in detail and the study just mentioned “the intervention program in times of COVID-19”	Mexico	Public	34 (I: 34)	Non-experimental, cross-sectional, exploratory-descriptive design	“Psychology for all” was based on brief psychotherapy, that is, cognitive behavioral treatment adding content under the humanistic-existential approach	Remote	Not described	Kessler	This study implemented an intervention program to reduce the aftereffects of the COVID-19 pandemic (implementation of “Psychology for All”) and measure mental health outcomes. Pre- and post-implementation comparisons of the implemented intervention program showed a decrease in the value of psychological distress.
[24]	Participants were recruited in July and August 2020	India	Primary caregivers of children with cancer	100 (I: 22)	Established existing intervention	Supportive psychotherapy based on humanistic psychology	Remote	Psycho-oncologist	PHQ GAD	Psychological distress was prospectively assessed via telephone calls using PHQ-9 and GAD-7 for the primary caregivers of pediatric cancer patients. Among high-risk individuals, GAD scores decreased after the intervention, whereas PHQ scores did not.
[25]	Participants were recruited between November 2020 and March 2021	Italy	Healthcare workers	225 (I: 68, C: 157)	Established existing intervention	Eye movement desensitization and reprocessing (EMDR) therapy	Remote	Psychologist/counselor	IES-R THERMO	This study investigated post-traumatic stress disorder in a sample of Italian healthcare workers during the COVID-19 outbreak and evaluated the efficacy of EMDR therapy on this population. The results showed that the IES-R and THERMO values decreased after the intervention in a pre- and post-intervention comparison of the EMDR treatment group only.
[26]	The details were not described	Canada, UK-based with multiple countries, USA, China	Public, staff, patients with COVID-19	32 (Canada), 646 (UK-based with multiple countries), 24 (USA), 235 (China)	Verification of feasibility, RCT, established and existing intervention	Virtual reality	Not described	Not described	Satisfaction etc.	This systematic review evaluated the role of virtual reality as a psychological intervention tool for mental health problems during the COVID-19 pandemic. Virtual reality is a beneficial tool for intervention in individuals with mental health problems.
[27]	Participants were enrolled between February 6 and March 9, 2020	China	Patients with COVID-19	67 (I: 67)	Established existing intervention	S-CBTI	Remote (patients were asked to participate in behavioral therapy by mobile phone and paper-based systems)	Medical doctors	ISI	This study developed a brief CBT for insomnia (S-CBTI) in patients with COVID-19 and comorbid insomnia symptoms and tested its efficacy in a self-controlled study. The second objective was to compare the effectiveness of S-CBTI between acute and chronic insomnia in women with COVID-19 and comorbid insomnia symptoms. The ISI scores in the intervention group improved before and after the intervention. After the intervention, the mean ISI score of the acute insomnia group was lower than that of the chronic insomnia group. The decrease in the ISI scores and improvement in sleep duration from baseline to post-intervention was greater in the acute insomnia group than in the chronic insomnia group.
[28]	Not described in detail; participants were recruited through a participant database hosted by the medical school	Singapore	Healthcare workers during the COVID-19 pandemic	80 (I: 40, C: 40)	Established existing intervention	Mobile app-based mindfulness	Mobile app	Not described	PCL, DAS, FCV-19S, PWI, ProQOL, PSQ, FFMQ, SCS, and digit span Tests (forward and backward)	This study investigated the effects of a mindfulness practice delivered using Headspace on the psychological and cognitive outcomes of healthcare workers in Singapore. The intervention improved the fear of COVID-19, compassion satisfaction, trait mindfulness, self-compassion, sleep quality, and forward digit span task at the 1-month follow-up.
[29]	Patients with COVID-19 were recruited from June to August 2020	Indonesia	Patients with COVID-19	47 (I: 42)	Verification of feasibility	Video-based psychotherapy including relaxation, management of thoughts and emotions, and mindfulness	Watching videos	The video (the creators were psychiatrists)	SUDS	This study investigated the effectiveness of video-based psychotherapy in reducing distress in COVID-19 patients being treated in an isolation ward. After the intervention, the SUDS scores decreased in the intervention group.
[30]	The program ran between March 11, 2019 and July 31, 2020	Australia	Public	10894 [pre-COVID: 2321, during-COVID: 8573] (I: 6132 [pre-COVID: 5074, during-COVID: 1058])	Verification of feasibility	Internet CBT	Remote	Medical doctors (and Internet CBT program)	Kessler PHQ GAD	This study assessed the uptake and effectiveness of Internet CBT for symptoms of anxiety and depression during the pandemic and compared outcomes with the 12 months before COVID-19. The Internet CBT course was effective at reducing anxiety, depression, symptom severity, and psychological distress both before and during the pandemic.
[31]	Participants were enrolled from March to June 2020	Italy	Patients with COVID-19	45 (I: 45)	Verification of feasibility	Tele psychotherapy (“Telecovid Sicilia”)	Remote	Psychologist/counselor	SCL-90-R, BDI, ESS, HARS.	This study investigated the effects of tele-psychotherapy on emotional well-being and psychological distress among patients affected by COVID-19. The intervention improved depression, anxiety, paranoid ideation, and sleep disorders.
[32]	Not described in detail; when healthcare professionals fought COVID-19	China	Healthcare professionals	4 (I: 4)	Verification of feasibility	Mindfulness-based stress reduction and virtual reality therapy	Virtual reality therapy	Virtual reality	PHQ GAD AIS	This case study examined the effectiveness of virtual reality therapy in the treatment of psychological problems. Four cases of front-line healthcare providers who presented with clinically significant initial psychological problems at the onset of COVID-19 and received virtual reality therapy treatment were described. After sessions, all four cases showed lower scores and improved symptoms for depression, anxiety, psychosomatic symptoms, and sleeping symptoms.
[33]	Participants were recruited in April and May 2020; the intervention run from April to November 2020	Italy	Patients with dementia and their caregivers	50 (caregiver group: 23, patient group: 25).	RCT	Telephone support	Remote	Psychologist/counselor and master’s level licensed social workers	NPI, ZBI, QOL	This study was conducted during the first lockdown period of COVID-19 to provide tele-psychological support to dementia patient caregivers and evaluate its effectiveness by quantifying stress load and quality of life. Caregivers who received telephone support with mood and stress burdens did not experience a worsening of their psychological state during the intervention period.
[34]	Not described in detail just mentioned “during the COVID crisis”	USA	Clinic clients	4 (I: 4)	Established existing intervention	CBT strategies	Remote	Psychologist/counselor	Fear, sadness, general distress	This study described the strategy of using CBT for clients with pandemic-related distress at a clinic during the COVID-19 pandemic. A case of a preliminary intervention was presented in which clinic clients’ fear, sadness, and general distress were reduced.
[35]	Details were not described (see measures section below; Tennant et al., 2007)	UK	Public	48 (I: 48)	RCT	ACT	Weekly online semi-interactive, self-help modules and a webinar	Not described	SWEMWBS, CompACT, DASS	This study examined the effectiveness of a guided self-help ACT intervention on well-being for the general population with COVID-related distress and explored the effects of psychological flexibility and distress as well as COVID-19 distress. It showed improvements in well-being, overall psychological flexibility (including the subscales of behavioral awareness and valued action), and general psychological distress (including depression, anxiety, and stress). However, no changes were found for COVID-related distress.
[36]	Details were not described	Canada	Temporary migrant live-in caregivers	46 (I: 18; C: 18)	RCT	ACT	Remote	Not described	DAS, AAQ, CAMS, MSMR	This study evaluated the effectiveness of the online delivery of a six-week psychological intervention based on ACT in reducing psychological distress and promoting resilience among live-in caregivers. The intervention showed improvement in mindful qualities, external resilience, life satisfaction, and accessible support.
[37]	Data were collected between January and August 2020	USA	Primary caregiver of a child	12 (I: 12)	Verification of feasibility	Connecting and Reflecting Experience program	Remote	Psychologist/counselor, licensed clinical psychologists, social workers etc.	PHQ GAD GSRS	The study assessed treatment engagement, acceptability, and psychological distress among participants after the onset of COVID-19 in the adaptation of telehealth using the Connecting and Reflecting Experience program. Self-reported mood and anxiety symptoms decreased after 20 weeks of telehealth therapy.

Note. PCL: Post-traumatic Stress Disorder Checklist; HSCL: Hopkins Symptom Checklist; PHQ: Patient Health Questionnaire; GAD: Generalized Anxiety Disorder; HADS: Hospital Anxiety and Depression; IES-R: Impact of Event Scale Revised for Post-traumatic Stress; THERMO: Emotion Thermometer; ISI: Insomnia Severity Index; DAS: Depression, Anxiety, and Stress Scale-21; FCV-19S: Fear of COVID-19 Scale; PWI: Personal Well-being Index; ProQOL: Professional Quality of Life Scale; PSQ: Perceived Sleep Quality; FFMQ: Five Facet Mindfulness Questionnaire; SCS: Self-Compassion Scale; SUDS: Subjective Units of Distress Scale; SCL-90-R: The Symptom Checklist-90-R; BDI: Beck Depression Inventory; ESS: Epworth Sleepiness Scale; HARS: Hamilton Anxiety Rating Scale; AIS: Athens Insomnia Scale; NPI: Neuropsychiatric Inventory; ZBI: Zait burden Interview; QOL: Quality of Life caregiver; SWEMWBS: Warwick-Edinburgh Mental Wellbeing short form; CompACT: the Comprehensive Assessment of Acceptance Commitment Therapy Processes, DASS: The Depression Anxiety Stress Scale; AAQ: Acceptance and Action Questionnaire; CAMS: Cognitive and Affective Mindfulness Scale; MSMR: Multi-System Model of Resilience; GSRS: Group Session Rating Scale.

Bars indicate the distribution (%) of target papers (n =  18). The number next to the bars indicates the number of papers that match each item.

a) Intervention participants.

The figure indicates the number of papers corresponding to each item: patients with COVID-19, patients who had recovered from COVID-19, caregivers, healthcare workers, and others. Others (n =  2) included clients at the clinic and refugees.

b) Types of psychotherapies and psychological support.

The figure indicates the number of papers corresponding to each item: humanistic psychology/psychological support, virtual reality therapy, telephone support, mindfulness, ACT, CBT, etc. Others (n =  4) included problem management plus EMDR therapy, parenting intervention, and video-based psychotherapy.

c) Types of interventions.

The figure indicates the number of papers corresponding to each item: feasibility validation, RCT, established existing intervention, and others. Others (n =  1) included an exploratory-descriptive design.

d) Means of psychotherapies and psychological support.

The figure indicates the number of papers corresponding to each item: remote, face-to-face, and others. Others (n =  6) included no intervention by any person but by a mobile app, educational video, watching virtual reality therapy, webinars, paper-based system (material), and no description.

e) Providers.

The figure indicates the number of papers corresponding to each item: psychologists/counselors, medical doctors, and others. Others (n =  11) included trained but non-specialist (n =  1), psycho-oncologist (n =  1), educational program (n =  3), “postdoctoral fellows, predoctoral psychology interns, and social workers” (n =  1), and not described (n =  5).

### Intervention participants

The participants in the studies included in this review were categorized as follows: patients with COVID-19 (n =  4, 22%) [26,27,29,31], patients who had recovered from COVID-19 (n =  1, 6%) [16], citizens (n =  5, 28%) [21,23,26,30,35], caregivers (n =  4, 22%) [24,33,36,37], and healthcare workers (n =  4, 22%) [25,26,28,32]. Moreover, there were two cases falling under the category of others: clients at a clinic (n =  1) [34] and refugees (n =  1) [20].

### Countries

The countries were widely distributed across Europe, Central and South Asia, East Asia, North America, and Central America (see Table 2).

**Table 2:** Countries of Psychotherapies and Psychological Support in the Reviewed Papers.

Region	Country	No. of papers
Middle East	Jordan	1
	Oman	1
Europe	Italy	3
	United Kingdom	2
North America	Canada	2
	United States	3
Central America	Mexico	1
East Asia	China	3
South Asia	India	1
	Singapore	1
	Indonesia	1
Oceania	Australia	1

### Types of psychotherapies and psychological support

The distribution of psychotherapies and psychological support was as follows: humanistic psychological support (n =  3, 17%) [22–24], virtual reality therapy (n =  2, 11%) [26,32], telephone support (n =  2, 11%) [31,33], mindfulness (n =  3, 17%) [28,29,32], ACT (n =  3, 17%) [21,35,36], CBT (n =  4, 22%) [21,27,30,34], and others (n =  4, 22%). Others included problem management plus (n =  1) [20], EMDR therapy (n =  1) [25], parenting intervention (n =  1) [37], and video-based psychotherapy (n =  1) [29] (see Fig 2).

### Types of interventions

The distribution of the types of interventions was as follows: validation of feasibility (n =  6, 33%) [26,29–32,37], RCT (n =  6, 33%) [20,21,26,28,33,36], established existing intervention (n =  7, 39%) [22,24–27,34,35], and others (n =  1, exploratory-descriptive design, 6%) [23] (see Fig 2).

### Means of psychotherapies and psychological support

The distribution of the means of psychotherapies and psychological support was as follows: remote (n =  11, 61%) [21,23–25,27,30,31,33,34,36,37], face-to-face (n =  2, 11%) [20,22], and others (n =  6, 33%) [26–29,32,35]. Others included a paper-based system [27], mobile app [28], educational video [29], watching virtual reality [32], webinar [35], and no description [26] (see Fig 2).

### Providers

The distribution of the types of providers was as follows: psychologists/counselors (n =  7, 39%) [21,22,25,31,33,34,37], medical doctors (n =  2, 11%) [27,30], and others (n = 11, 61%). The majority fell under the category of others and the breakdown was as follows: trained but non-specialist (n =  1) [20], psycho-oncologist (n =  1) [24], not human but educational program (n =  3) [29,30,32], postdoctoral fellows (n =  1) [37], post/predoctoral psychology interns (n =  1) [37], social workers (n = 1) [37], and not described (n =  5) [23,26,28,35,36] (see Fig 2).

### Means of the measures used

The means of measuring the outcomes of the interventions were as follows: depression (Kessler, Beck Depression Inventory), anxiety (Generalized Anxiety Disorder, Hamilton Anxiety Rating Scale), depression and anxiety (Hospital Anxiety and Depression Scale, Hopkins Symptom Checklist, Depression, Anxiety, and Stress Scale), post-traumatic stress (Post-traumatic Stress Disorder Checklist, The Impact of Event Scale － Revised), sleep (Insomnia Severity Index, Athens Insomnia Scale, Epworth Sleepiness Scale, Perceived Sleep Quality), and physical symptoms and general health (Patient Health Questionnaire). The following assessments were performed: Emotion Thermometer, Fear of COVID-19 Scale, Personal Well-being Index, Professional Quality of Life Scale, Subjective Units of Distress Scale, Neuropsychiatric Inventory, Five Facet Mindfulness Questionnaire, Self-Compassion Scale, Digit Span Tests (Forward and Backward), Symptom Checklist-90-R, Zait Burden Interview, Quality of Life Caregiver, Warwick-Edinburgh Mental Wellbeing short form, Comprehensive Assessment of Acceptance Commitment Therapy Processes, Acceptance and Action Questionnaire, Cognitive and Affective Mindfulness Scale, Multi-System Model of Resilience, and Group Session Rating Scale (see Table 1).

## Discussion

In this study, we investigated the types of psychotherapies and psychological support provided during COVID-19, target recipients, and delivery methods employed. When a disaster (including an outbreak of an infectious disease) occurs, the call for psychological support is often loud. In this research, we took a broad view of the types of psychotherapies and psychological support provided following the global scourge of COVID-19 and offer a number of novel findings.

For the target population, a wide range was found, including the public [21,23,26,30,35], caregivers [24,33,36,37], healthcare workers [25,26,28,32], COVID-19 patients [26,27,29,31], and patients who had recovered from COVID-19 [22]. Reports of worsening mental health as a sequela of COVID-19 have been numerous worldwide [38]. In this study, the target population included all people, regardless of whether they had been infected with COVID-19 and whether they had post-COVID-19 sequelae. However, among the studies identified by this review, none specifically examined people with post-COVID-19 sequelae, although one study provided psychological support for people who had recovered from COVID-19, including those suffering from depression or anxiety [22]. The lack of studies on patients with post-COVID-19 sequelae may be owing to the difficulty in maintaining a post-discharge connection with patients in the chronic phase of symptoms, such as those in the sequelae (which also leads to difficulties in recruiting study participants). Psychiatric symptoms in the chronic phase are diverse and difficult for both patients and practitioners to identify as sequelae of COVID-19. Alternatively, most people with COVID-19 may have experienced spontaneous remission within approximately one year [39, 40] and it may have been difficult to recruit participants for clinical trials.

The regional spread of the COVID-19 pandemic was clear from our review findings, with psychotherapies and psychological support provided across regions. Further research is needed to determine the driving factors of the differences in the number of reports by country found in this study. It may have been that the chosen countries have environments conducive to the provision and receipt of psychotherapies and psychological support. Alternatively, it may have been the result of social demand, that is, people may have been seeking psychotherapies and psychological support for their psychological recovery.

The types of psychotherapies and psychological support varied from well-known therapy and support (i.e., support based on humanistic psychology, CBT, ACT, mindfulness) [21,22,24,27–30,32,34–36] to relatively new methods (i.e., virtual reality therapy) [26,32]. The majority of the means of conducting psychotherapies and psychological support were remote interventions rather than conventional face-to-face interventions [21,23–25,27,30,31,33,34,36,37]. This could have been attributed to the infectious nature of COVID-19. It may also be that sufficient technological innovation had already occurred for non-face-to-face psychotherapies and psychological support to be provided and that the COVID-19 disaster provided an opportunity for its implementation. This study’s results also hinted that the paradigm of the psychiatry world was influenced by the social situation during the COVID-19 pandemic; however, the review findings did not directly show whether social factors played a role. It remains to be seen whether the technologies and support methods rapidly introduced will take hold now the threat of COVID-19 has diminished.

Regarding the types of interventions, feasibility studies [26,29–32,37,41], RCTs [20,21,26,28,33,36], and existing interventions were established [22,24–27,34,35]. Although the majority of the studies in this review provided online psychotherapies and psychological support, only three studies involved online RCTs [21,33,36]. Moreover, no studies compared the effectiveness of online/remote psychotherapies and psychological support with face-to-face support. Therefore, further research on this topic is required.

In this scoping review, interventions by professionals dealing with psychotherapies and psychological support (medical doctors and certified psychotherapists/psychologists) did not account for the majority [21,22,25,27,30,31,33,34,37]. This suggests the difficulty in obtaining support from psychiatric professionals, replacement by other professionals or novel technologies, or the difficulty for physicians and psychologists to conduct large-scale interventions and publish the results because of their budget and human resources constraints, except in some of the most advanced regions. The replacement of psychiatric services with new technology (i.e., apps) offers several advantages to service recipients. First, it enhances accessibility to psychotherapies and psychological support, reducing barriers that may hinder individuals from seeking assistance. Second, this approach can lead to the standardization and homogenization of such support, which vary in quality among therapists. Regarding outcomes, a range of mental health-related items were employed, including anxiety, post-traumatic stress, sleep, and the fear of COVID-19 scale in addition to the primary focus on distress. Notably, most studies used multiple outcomes, suggesting a comprehensive assessment of participants’ mental health status to provide robust support.

This study clarified to whom and by what means psychotherapies and psychological support was provided during the COVID-19 pandemic, considering the characteristics of COVID-19 as an infectious disease, social context of the pandemic, and methods and means by which it was selected and implemented. However, this scoping review also revealed the following research gaps. First, the implementation of psychotherapies and psychological support for patients with post-COVID-19 sequelae may have been insufficient (patients may have been overlooked). Second, it is necessary to clarify the relative effectiveness of online/remote and face-to-face RCTs during the pandemic. Third, it is unclear whether the technology used during the pandemic will continue to be used. Lastly, research is needed on how physicians, psychologists, and other psychiatric professionals differentiate and coexist with non-human technologies (i.e., bots and apps). These issues need to be closely monitored in the long term.

### Limitations

The first limitation of this study is the number of search databases used (i.e., two). Tricco et al. [16] state, “Present the full electronic search strategy for at least one database, including any limits used, such that it could be repeated.” In other words, the method used in this study meets this guideline. However, using more databases may have opened up access to more papers. Additionally, the papers included in this review are limited to those written in English. In other words, the data available for the study are limited.

Second, the selection of psychotherapies and psychological support may have been influenced by factors such as the ease of implementation and availability of evidence-based measures. Therefore, this approach may not fully encompass the complexity and nuances of current clinical situations.

Finally, in this study, telephone support is treated as one type of support since the content of telephone support is not always classifiable in the psychiatric or clinical psychology field.

## Conclusions

This study revealed the countries in which psychotherapies and psychological support were implemented during the COVID-19 pandemic, the main providers, and the major means of implementation. The following points are noteworthy:

- No studies on the use of psychotherapy for patients suffering from the sequelae of COVID-19 were found.
- The implementation of psychotherapy was not limited to more advanced countries in psychiatry, indicating the broader reach of these interventions.
- Psychotherapy includes many methods, including CBT, ACT, mindfulness, supportive care, virtual reality therapy, and online educational content via apps. The rise of new technologies may have increased the replacement rate of human therapists.
- The majority of psychotherapy interventions were delivered remotely. However, whether this trend will persist beyond the COVID-19 pandemic remains uncertain. Further research on the differences in effectiveness between remote and face-to-face psychotherapy is needed.
- Finally, most of those providing psychotherapies and psychological support in the studies included in this review were not doctors (n = 2, 11%) or psychologists (n = 7, 39%).

## Supporting information

S1 Table List of target papers.
(XLSX)
